# Distinct severity of phenotype in Hajdu-Cheney syndrome: a case report and literature review

**DOI:** 10.1186/s12891-020-3181-0

**Published:** 2020-03-06

**Authors:** Chunhua Zeng, Yunting Lin, Zhikun Lu, Zhen Chen, Xiaoling Jiang, Xiaojian Mao, Zongcai Liu, Xinshuo Lu, Kangdi Zhang, Qiaoli Yu, Xiaoya Wang, Yonglan Huang, Li Liu

**Affiliations:** 1 Department of Genetics and Endocrinology, Guangzhou Women and Children’s Medical Center, Guangzhou Medical University, 9 Jinsui Rd, Guangzhou, 510623 China; 2 Department of Radiology, Guangzhou Women and Children’s Medical Center, Guangzhou Medical University, 9 Jinsui Rd, Guangzhou, 510623 China; 3 Department of Dentistry, Guangzhou Women and Children’s Medical Center, Guangzhou Medical University, 9 Jinsui Rd, Guangzhou, 510623 China; 4 Department of Otolaryngology, Guangzhou Women and Children’s Medical Center, Guangzhou Medical University, 9 Jinsui Rd, Guangzhou, 510623 China

**Keywords:** *NOTCH2*, Hajdu-Cheney syndrome, Osteoporosis, Acro-osteolysis

## Abstract

**Background:**

Hajdu-Cheney syndrome (HCS) is a rare inherited skeletal disorder caused by pathogenic mutations in exon 34 of *NOTCH2*. Its highly variable phenotypes make early diagnosis challenging. In this paper, we report a case of early-onset HCS with severe phenotypic manifestations but delayed diagnosis.

**Case presentation:**

The patient was born to non-consanguineous, healthy parents of Chinese origin. She presented facial anomalies, micrognathia and skull malformations at birth, and was found hearing impairment, congenital heart disease and developmental delay during her first year of life. Her first visit to our center was at 1 year of age due to cardiovascular repair surgery for patent ductus arteriosus (PDA) and ventricular septal defect (VSD). Skull X-ray showed wormian bones. She returned at 7 years old after she developed progressive skeletal anomalies with fractures. She presented with multiple wormian bones, acro-osteolysis, severe osteoporosis, bowed fibulae and a renal cyst. Positive genetic test of a de novo heterozygous frameshift mutation in exon 34 of *NOTCH2* (c.6426dupT) supported the clinical diagnosis of HCS.

**Conclusion:**

This is the second reported HCS case caused by the mutation c.6426dupT in *NOTCH2,* but presenting much earlier and severer clinical expression. Physicians should be aware of variable phenotypes so that early diagnosis and management may be achieved.

## Background

Hajdu-Cheney syndrome (HCS; OMIM 102500) is an extremely rare and heterogeneous disease that can be characterized by craniofacial anomalies, acro-osteolysis, progressive osteoporosis with fractures, congenital heart defects, hearing impairment, polycystic kidneys, short stature and developmental delay [1–3]. HCS exhibits autosomal dominant inheritance, but there have been several sporadic cases previously reported. Truncating mutation in Notch homolog protein 2 gene (*NOTCH2*) was reported as the main cause of HCS [4–6]. The *NOTCH2* gene, located on 1p12-p11 (NM_024408.3), encodes a transmembrane protein critical in skeletal development and bone remodeling by acting on the cells of osteoclast and osteoblast lineage [7]. The *NOTCH2* mutation is a gain of function, as it results in a longer half-life for NOTCH2 protein [8, 9]. The enhanced NOTCH2 activity promotes osteoclastogenesis and inhibits osteogenesis.

To date, fewer than 100 HCS cases have been described in medical literature, with only few cases regarding the Chinese population [10, 11]. The clinical manifestation in HCS is highly variable, which makes early diagnosis challenging, resulting in delayed diagnosis in many cases [12, 13]. Here, we report a Chinese girl with HCS who exhibited significant phenotypes during her first year of life, but was only diagnosed at the age of 7 years after developing severe osteoporosis with fractures.

## Case presentation

### Patient’s characteristics

The patient, a seven-year-old Chinese girl, was the first child born to healthy non-consanguineous parents. Her birth weight was 3200 g. She was found facial anomalies including small mouth, flat nasal base, long philtrum and micrognathia at the age of few months. No positive family history was noted.

### Clinical and radiological findings

At one year old, the patient was found to have a ventricular septal defect (VSD), patent ductus arteriosus (PDA), pulmonary hypertension and developmental delay after experiencing several episodes of pneumonia. The patient also had an open anterior fontanelle (0.5 cm × 1.0 cm) and focal defects of the occipital bones. Radiographs showed normal arms and fingers (Fig. 2e). Computed Tomography (CT) Scan of the head revealed wormian cranial bones and patent cranial sutures (Fig. 2a). Magnetic resonance imaging (MRI) of the brain was interpreted as normal. At 1 year and 2 months, a successful repair surgery for congenital heart defects was done. Hearing impairment, progressive skeletal anomalies, gross motor and verbal delay and short stature were subsequently noticed by the parents. However, the patient did not undergo further examination until she was 5 years old, during which she presented developmental delay and facial dysmorphism. Her serum alkaline (ALP), calcium and phosphorus levels were all within normal range at the time (Table 1).

**Table 1:** Osteologic characterization of the HCS patient during 6 year follow-up

Parameter	1 year old	5 year old	7 year old	Normal range
Serum ALP (U/l)	112.0	313.0	558.0	118.0–390.0
Serum Calcium (mmol/L)	2.2	2.3	2.4	2.2–2.7
Serum Phosphorus (mmol/L)	–	1.2	1.7	1.3–1.9
Serum PTH (pmol/L)	–	–	2.7	1.2–7.1
Serum BALP (U/l)	–	–	240.0	0–200.0
Serum 25(OH) D (nmol/L)	–	–	66.0	50.0–150.0

*ALP* alkaline phosphatase, *BALP* bone alkaline phosphatase, *PTH* Parathyroid hormone, *25(OH) D* 25-hydroxyvitamin D

**Fig. 1:**
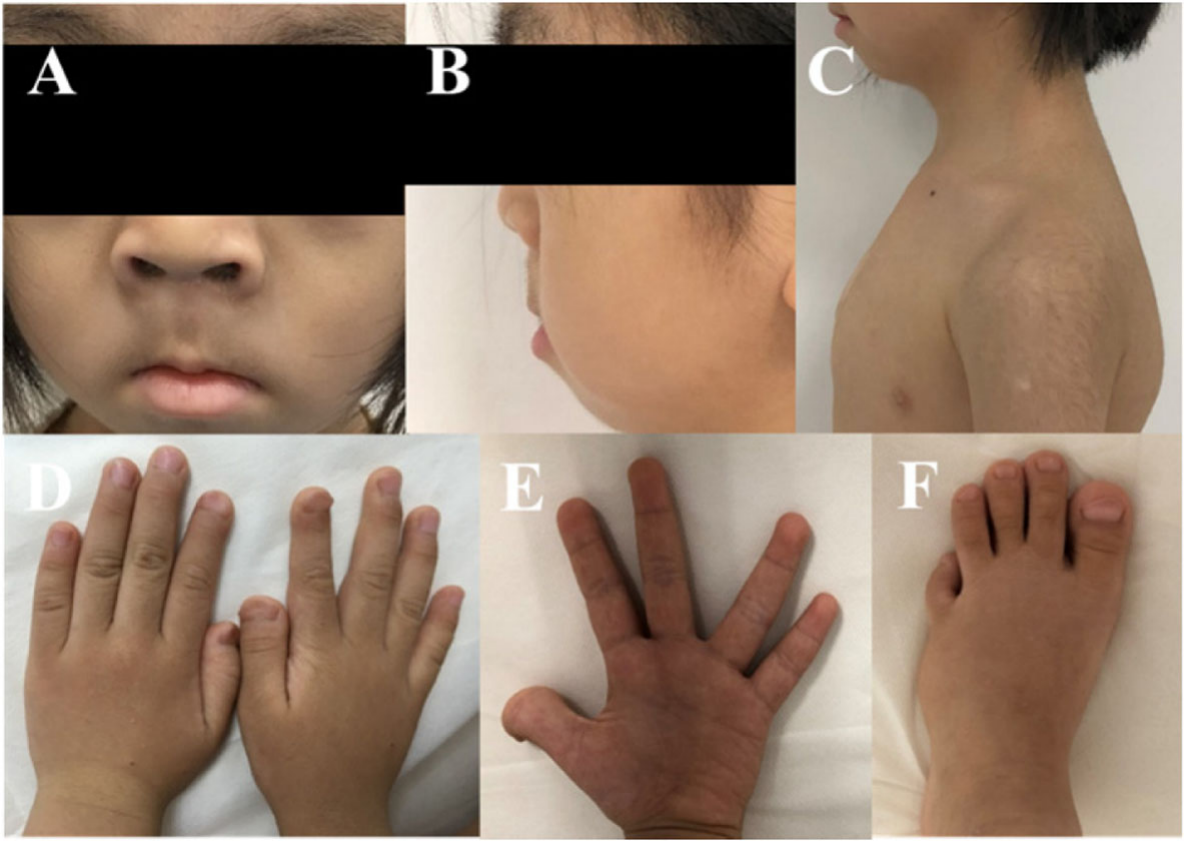
Photographs of the patient at 7 years of age. **a** and **b** Facial anomalies include coarse and thick hair, wide and arched eyebrows, a flat and broad nasal base, a long philtrum and micrognathia; **c** Photographs show thick body hair and a dysmorphic sternum; **d** and **e**) Shortened fingers and wide nails with pseudo-clubbing aspect; **f** Dysmorphic metatarsal bones and a shortened left toe

**Fig. 2:**
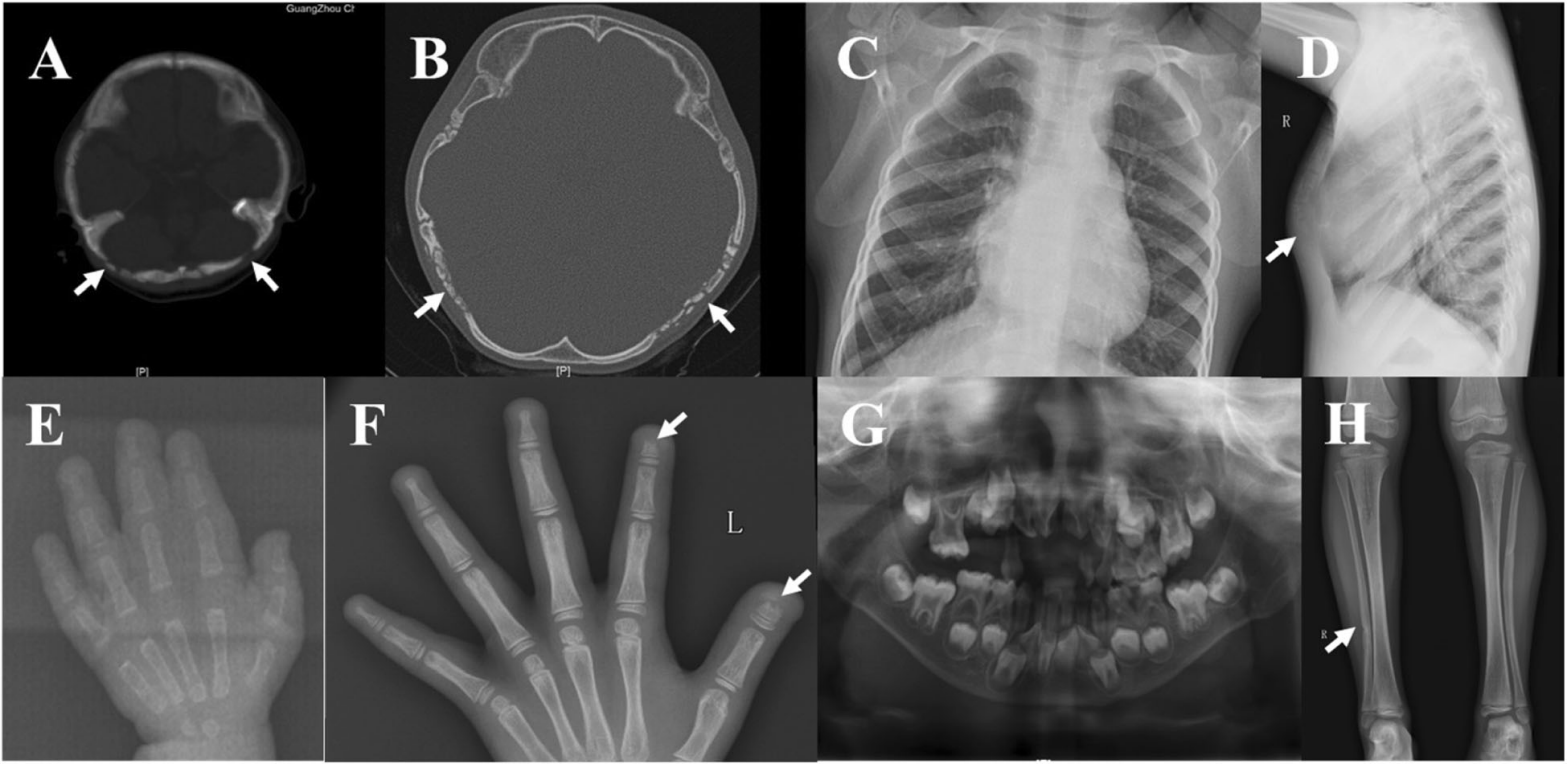
Computed Tomography (CT) scan and radiographs. **a** and **b** Wormian bones (arrow) and patent cranial sutures at 1 year and 2 months of age and at 7 years of age, respectively; **c** and **d** Wormian bones and deformity of the sternum at 7 years of age (arrow); **e** and **f** Acro-osteolysis was not present at 1 year and 2 months of age but was present in the distal phalanges at 7 years of age (arrow); **g** Misaligned and abnormally shaped teeth with dental malocclusion at 7 years of age; **h** Radiographs exhibiting bowed fibulae at 7 years of age (arrow)

The patient came back to our clinic at the age of 7 years due to experiencing metatarsal fractures twice within the past 3 months. She presented with distinct facial dysmorphism, multiple skeletal anomalies, hearing impairment and mild delay of mental development (Table 2). Her physical examination was significant for short stature with a height of 110.8 cm (<3rd percentile) and weight of 15.5 kg (<3rd percentile), a small mouth, coarse and thick hair, wide and arched eyebrows, a flat and broad nasal base, a long philtrum and micrognathia (Fig. 1a and b). In addition, she showed occipital skull defects, dysmorphic sternum, dysmorphic hands and feet, short fingers, wide nails and joint hyperlaxity (Fig. 1c-e). Her teeth were misaligned and abnormally shaped with dental malocclusion. Blood tests showed normal liver and renal function. Serum calcium, phosphate and parathyroid hormone levels were all in normal range. Serum ALP had kept increasing and reached to 558 U/L by the time she was 7 years old, which was much higher than the upper limit of normal range (Table 1). Serum bone ALP (BALP) was also higher than the upper limit of normal range. Urine was not collected and tested for bone resorption. A hearing test revealed bilateral conductive hearing impairment with a threshold of 48 dB in the right ear and 55 dB in the left ear. Head CT exhibited multiple wormian bones in the skull (Fig. 2b). Radiographs detected misaligned and abnormally shaped teeth with dental malocclusion, acro-osteolysis in distal phalanges of the fingers, wormian bones in the dysmorphic sternum, curved fibulae and a shortened left toe (Fig. 2c, d, f-h). Abdominal ultrasonography revealed a 5 mm × 4 mm renal cyst. Ultrasound bone sonometer detected low bone mineral density (BMD) in the long bones.

**Table 2:** Clinical features of two HCS patients carrying mutation c.6426dupT of *NOTCH2*

The proband	The case in the literature	The patient in current study	
Gender	Male	Female	
Age (yrs)	19-year-old	1-year-old	7-year-old
Craniofacial features			
Facial dysmorphology	**+**	**+**	**+**
Micrognathia	**–**	**+**	**+**
Periodontal disease	**+**	**–**	**+**
Cognitive/sensory function			
Developmental delay	**–**	**+**	**+**
Neurologic symptoms	**+**	**+**	**+**
Hearing deficit	**+**	**+**	**+**
General physical features			
Short stature	**+**	**+**	**+**
Congenital heart defect	PDA, VSD	PDA, VSD	repaired CHD
Polycystic kidneys	**–**	**–**	**+**
Joint hyperlaxity	**+**	**+**	**+**
Radiographic abnormalities			
Acroosteloysis	**+**	**–**	**+**
Osteoporosis	**+**	**–**	**+**
Wormian bones	**+**	**+**	**+**
Bowing of the fibula	**–**	**–**	**+**
Vertebral compression	**–**	**–**	**–**
Additional features			
Dysmorphic sternum	**–**	**–**	**+**
Metatarsal fracture	**–**	**–**	**+**

*PDA* patent ductus arteriosus, *VSD* ventricular septal defect, *CHD* congenital heart disease

### Genetic analysis

**Fig. 3:**
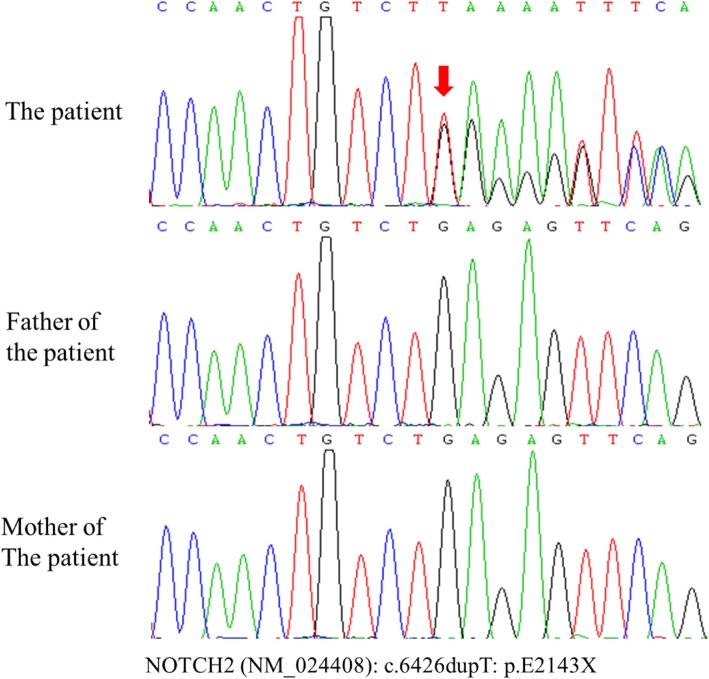
Chromatograms of *NOTCH2* mutations identified in the patient. The patient is a heterozygous carrier with a de novo duplication at position 6426 of the *NOTCH2* gene (exon 34)

After obtaining informed consent, genomic DNA was extracted from peripheral leukocytes of the patient and her parents. Next-generation sequencing (NGS) identified a duplication at position 6426 of the *NOTCH2* gene (exon 34) (NM-024408, c. 6426dupT) (Fig. 3). Sanger sequencing subsequently confirmed the mutation as de novo in the proband but not in her parents. The truncating mutation of c.6426dupT was reported as a pathogenic variant causing a frameshift change (p.E2143X).

## Discussion and conclusion

HCS is a rare autosomal dominant disorder caused by a gain-of-function mutation in the gene *NOTCH2*. A total of 85 mutations have been reported, 55 of which are missense mutations. In a literature review of reported HCS cases, all *NOTCH2* mutations are dispersed throughout the exon 34, and the most common mutation p.Q2208X was found in only 4 of the 36 families [14]. The clinical manifestations in reported HCS patients are highly variable, including facial dysmorphia, osteoporosis, acro-osteolysis, hearing impairment and dental implication [15]. Phenotypic variability resulted in diagnostic challenges and caused difficulty in predicting the clinical outcome from a specific genotype in HCS [15].

In this 7-year-old Chinese girl, we identified a de novo truncating mutation (c.6426dupT) in exon 34 of *NOTCH2*. The patient demonstrated the majority of clinical manifestations of HCS reported in previous studies. She presented with facial dysmorphia at birth, and as early as 1 year and 2 months old, she was found with cranial wormian bones, congenital heart disease and developmental delay. Chromosomal anomaly was suspected and then ruled out by karyotype analysis. After reparative surgery for PDA and VSD, the patient developed hearing impairment, periodontal disease and multiple skeletal anomalies in the following years. However, HCS was not diagnosed in this case until the patient had demonstrated severe osteoporosis with fractures, acro-osteolysis, renal cyst and significant developmental delay at the age of 7 years.

Our report demonstrates that the mutation c.6426dupT in this patient manifested with early-onset phenotype and severe clinical expressions with progressive acro-osteolysis and osteoporosis, hearing impairment and renal cyst. A literature review indicated this is the second case with this mutation; the first was previously reported in a 19-year-old male with HCS [4]. To further investigate the phenotype of the c.6426dupT mutation, we compared our patient to the reported case in the literature. Table 2 summarizes clinical and radiological manifestations in the two patients with the mutation of c.6426dupT in *NOTCH2*. We found both patients to exhibit facial dysmorphia, periodontal disease, hearing deficit, short stature, congenital heart disease (PDA and VSD), acro-osteolysis, osteoporosis and wormian bones. Despite multiple systems being affected, the craniofacial and skeletal anomalies were shared characteristic manifestations in both patients. Comparatively, the patient in our study demonstrated more significant manifestations including micrognathia, bowing of the fibulae, metatarsal fracture and developmental delay, which was not described in the first reported case. Additionally, our patient presented most of her characteristic manifestations of HCS during her first year of life. These results suggest that HCS caused by the same causative mutation may present a broad range of clinical severity and distinct phenotypic expression, making both diagnosis and prognosis challenging.

HCS is associated with gain-of-function mutations. Recent studies have suggested that Notch2 inhibits osteoblast differentiation, and induces and enhances osteoclastogenesis [16–19]. A mouse model of HCS further supported that bone loss is secondary to increased osteoclastogenesis and bone resorption [16]. Another study indicated that HCS not only presents as increased osteoclastogenesis, but also displays activated osteoblast activity with high bone turnover [17]. Similarly, the patient in our study exhibits osteologic features with increased bone formation. After her first bone fracture, the patient was prescribed with calcium and Vitamin D, and then followed up at our clinic for 8 months. However, she was not given anti-resorptive treatment due to her age and the parents’ concerns.

In conclusion, the findings support that the mutation c.6426dupT is pathogenic. Our study demonstrated variable degrees of expression and distinct severity of phenotype amongst HCS patients carrying the same *NOTCH2* mutation. The variability of phenotype indicates that there may be other modifier acting on the expression of truncating mutations in exon 34 of the gene *NOTCH2.* Although the mechanism resulting in HCS is not fully understood, physicians should be aware of variable phenotypes so that early diagnosis and management can be achieved.

## Data Availability

Not applicable. All data concerning the case are presented in the manuscript.

## Abbreviations

CT: Computed tomography
HCS: Hajdu-Cheney syndrome
MRI: Magnetic resonance imaging
*NOTCH2*: Notch homolog protein 2 gene
PDA: Patent ductus arteriosus
VSD: Ventricular septal defect
